# Cystic Encephalomalacia in a Young Woman After Cardiac Arrest Due to Diabetic Ketoacidosis and Thyroid Storm

**DOI:** 10.7759/cureus.23707

**Published:** 2022-03-31

**Authors:** Cheng-Hsun Chang, Hung-Wei Lian, Yueh-Feng Sung

**Affiliations:** 1 Department of General Medicine, Tri-Service General Hospital, National Defense Medical Center, Taipei, TWN; 2 Department of Neurology, Tri-Service General Hospital, National Defense Medical Center, Taipei, TWN

**Keywords:** magnetic resonance imaging, thyroid storm, diabetic ketoacidosis, hypoxia, cystic encephalomalacia

## Abstract

Cystic encephalomalacia is commonly reported in neonates with prenatal or perinatal hypoxic events. It is rarely observed in adults. A 25-year-old woman with a history of type 1 diabetes mellitus and hyperthyroidism presented to the emergency department with diabetic ketoacidosis (DKA) and a thyroid storm. She sustained cardiac arrest due to ventricular fibrillation and subsequently developed hypoxic encephalopathy. Initial brain computed tomography showed no significant findings; however, follow-up magnetic resonance imaging three months later revealed cystic encephalomalacia in the bilateral parieto-occipital lobes. A Tc-99m ethyl cysteinate dimer (ECD) brain perfusion scan revealed extensive hypoperfusion in the bilateral frontal and parieto-occipital lobes. She showed severe cognitive impairment and marked spasticity in all her limbs. Although cystic encephalomalacia is mostly reported in neonates with hypoxic injury, it can be seen in adults with hypoxic encephalopathy, leading to a significant neurological deficit.

## Introduction

Cystic encephalomalacia is a variant of encephalomalacia in which varying-sized cystic lesions within the white matter and cortex are present as a result of an extensive brain insult. It is mostly reported in neonates who have undergone prenatal or perinatal hypoxic events, leading to irreversible neuronal damage [1]. While hypoxic-ischemic brain injury is the most common cause of cystic encephalomalacia in neonates [2], it can be a consequence of several etiologies, including viral encephalitis, head trauma, and severe congenital metabolic disorders [1,3]. In adults, although rare, cystic encephalomalacia can follow an acquired parenchymal brain injury such as infection [4], trauma [5], or infarction [6]. Here, we present a 25-year-old woman with cystic encephalomalacia caused by hypoxic brain injury.

## Case presentation

**Figure 1:**
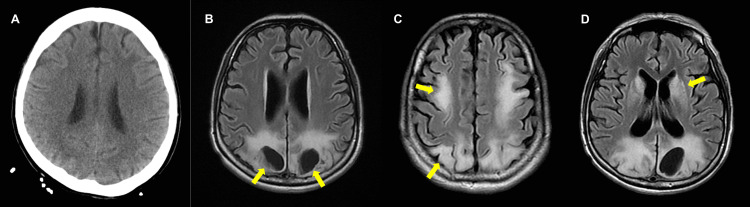
Computerized tomography of brain and T2-FLAIR brain magnetic resonance imaging Computerized tomography of brain performed 12 days after ROSC showing no remarkable findings (A). T2-FLAIR brain magnetic resonance imaging performed three months later revealing cystic encephalomalacia in bilateral parieto-occipital lobes (B), symmetrical T2-FLAIR hyperintense lesions, involving the bilateral fronto-parieto-occipital gray matter and white matter (C), basal ganglia (D), and splenium of the corpus callosum (not shown).

A 25-year-old woman with a history of type 1 diabetes mellitus and hyperthyroidism presented to the emergency department of a regional hospital with generalized malaise, epigastric pain, vomiting, and dyspnea. According to her family, she has a normal mentality but has been non-compliant with medications for six weeks. Blood tests were remarkable for glucose 761 mg/dL, blood ketone 2+, BUN 34 mg/dL, total bilirubin 1.8 mg/dL, AST 813 U/L, LDH 1628 U/L, free T4 2.48 ng/dL, and TSH <0.012 uIU/ml. Arterial blood gas showed a pH of 6.897, HCO_3_ of 4.1 mmol/L, and a base excess of −27.6 mmol/L. She was diagnosed with diabetic ketoacidosis (DKA) and a thyroid storm. Unfortunately, the patient developed ventricular fibrillation and cardiac arrest. Return of spontaneous circulation (ROSC) was achieved within 10 minutes after cardiopulmonary cerebral resuscitation; however, the patient was encephalopathic after ROSC was achieved and generalized seizures were also reported. An antiepileptic drug with levetiracetam, 2000 mg/day, was administered. DKA was treated with insulin and fluid. A thyroid storm was managed with propranolol, steroids, and propylthiouracil. She was successfully extubated seven days later. However, consciousness remained comatose. Brain computed tomography (CT) performed 12 days after ROSC showed no significant findings (Figure 1A).

Three months later, the patient was referred to our hospital for evaluation and management. Although she was awake, her cognitive function was severely influenced. She could only obey simple commands, such as raising her hands and answering simple questions by nodding or shaking her head. There was no verbal output. A neurological examination revealed spasticity, hyperactive deep tendon reflexes, and reduced muscle power (MRC grade 3) in all extremities. Primitive reflexes, such as snout, palmomental, and glabellar reflexes, were observed. Electroencephalography revealed no normal alpha background activity and increased beta activity. Brain magnetic resonance imaging (MRI) revealed cystic encephalomalacias in the bilateral parieto-occipital lobes (Figure 1B). There were also symmetrical T2-FLAIR hyperintense lesions involving the bilateral fronto-parieto-occipital gray matter and white matter, basal ganglia, and splenium of the corpus callosum, compatible with hypoxic brain injury (Figure 1C-1D).

**Figure 2:**
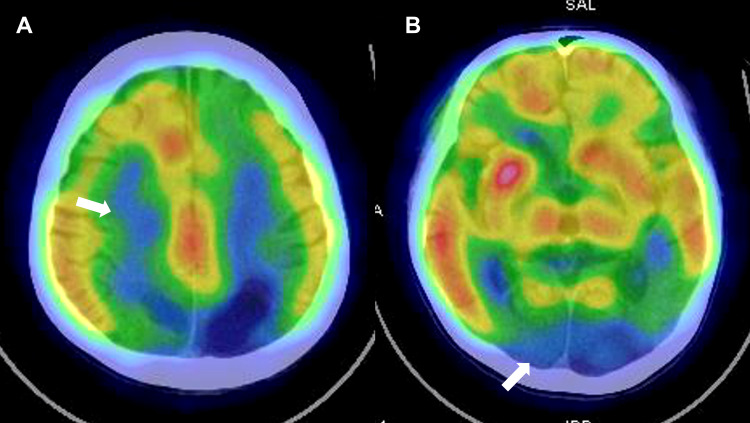
Tc-99m ECD brain perfusion scan Tc-99m ECD brain perfusion scan showing extensive hypoperfusion over bilateral frontal and parieto-occipital lobes (A, B).

A Tc-99m ethyl cysteinate dimer (ECD) brain perfusion scan revealed extensive hypoperfusion in the bilateral frontal and parieto-occipital lobes (Figure 2A-2B).

She underwent a rehabilitation program and a focal injection of botulinum toxin for limb spasticity. Hyperbaric oxygen therapy was administered, but the cognitive impairment did not improve. At her last follow-up at our outpatient department (eight months after hypoxic brain injury), her neurological deficit was unchanged.

## Discussion

Hypoxic brain injury after cardiac arrest is a significant cause of mortality and long-term disability. The injury may be focal, diffuse, or global, with the degree of severity ranging from transient brain edema to irreversible brain infarction and necrosis [7]. Imaging plays an important role in the diagnosis of the severity of brain damage. Early brain CT obtained within 24 hours of cardiac arrest may be unremarkable or show only subtle hypoattenuation of the deep gray matter structure [8]. Subsequent CT will show diffuse basal ganglia abnormalities along with diffuse cerebral edema, manifesting as cortical hypoattenuation, loss of normal gray-white matter differentiation, and effacement of the sulci, ventricles, and basal cisterns [7,8]. The MRI findings of hypoxic encephalopathy differ between neonates and adults. In the postnatal period, severe hypoxic insults result in diffuse gray matter damage, with relative sparing of the perirolandic cortex and regions supplied by the posterior circulation. However, the deep gray matter nuclei, cortices, hippocampi, and cerebellum are mostly affected in older children and adults after severe hypoxic-ischemic injury [8]. Cystic encephalomalacia is commonly seen in perinatal hypoxic encephalopathy [1].

We present the case of a young woman who experienced metabolic derangements secondary to DKA and a thyroid storm. It is well known that both DKA and thyroid storms can cause life-threatening arrhythmias, cardiac arrest, and subsequent hypoxic-ischemic brain injury. However, DKA, or the thyroid storm, primarily can cause irreversible brain damage. Previous studies have shown that DKA in patients with diabetes 1 results in morphologic and functional brain changes that are associated with adverse neurocognitive outcomes [9]. Another study analyzed comas in patients with thyroid storms. In that study, the authors attributed the brain damage to alterations in brain hemodynamics, energy metabolism, neurotransmitters, and brain network connectivity [10]. Therefore, we hypothesized that hypoxic encephalopathy with cystic encephalomalacia in our patient may be attributed to hypoxic brain injury combined with metabolic derangements. To the best of our knowledge, there are no previous reports of adult-onset cystic encephalomalacias caused by hypoxic-ischemic insult secondary to cardiac arrest. It is not clear why some children develop cystic encephalomalacia after ischemic brain injury, whereas adults do not. Further research is needed to delineate the pathomechanism. Like infancy, adults with hypoxic brain injury with cystic encephalomalacia can lead to severe neurological deficits and predict a poor prognosis.

## Conclusions

While cystic encephalomalacia is found mostly in neonates who have undergone prenatal or perinatal hypoxic events, it can also be seen in adults after hypoxic brain injury and metabolic derangements. Cystic encephalomalacia in adults can lead to a severe neurological deficit and predict a poor outcome.
